# Can crabs kill like a keystone predator? A field-test of the effects of crab predation on mussel mortality on a northeast Pacific rocky shore

**DOI:** 10.1371/journal.pone.0183064

**Published:** 2017-08-24

**Authors:** Wesley W. Hull, Paul E. Bourdeau

**Affiliations:** 1 Telonicher Marine Laboratory, Humboldt State University, Trinidad, California, United States of America; 2 Department of Biological Sciences, Humboldt State University, Arcata, California, United States of America; University of California, UNITED STATES

## Abstract

Predation can strongly influence community structure and ecosystem function, so the loss of key predators can have dramatic ecological consequences, unless other predatory species in the system are capable of playing similar ecological roles. In light of the recent outbreak of sea star wasting disease (SSWD) and subsequent depletion of west coast sea star populations, including those of the keystone predator *Pisaster ochraceus*, we examined whether large mobile crabs could play a role as predators on mussels (*Mytilus californianus*) on a rocky shore in Northern California. Using a combination of sea star removal and predator exclusion cages we found that mussel mortality was 43–294 times greater in uncaged treatments versus caged treatments. Mortality on uncaged mussels at low tidal elevations was due to predation by large mobile crabs (*Cancer productus* and *Romaleon antennarium*); confirmed by the presence of mussel shell fragments and documented attacks on wax snail replicas. Laboratory feeding assays indicated that crabs, on a per unit biomass basis, can consume almost twenty-five times as many mussels per day than sea stars, which together with the results of our field experiment, suggest that large predatory crabs could play an important role in maintaining ecosystem function through their predation on mussels on rocky shores where *P*. *ochraceus* are rare, absent, or have been depleted by SSWD.

## Introduction

Understanding the role of species redundancy and/or compensation within ecosystems will be critical for predicting the impacts of increasing local extinctions in a rapidly changing global environment. Because individual species can play critical ecological roles, e.g., keystone species [1–4], the loss of these species via extinction can cause major changes in ecosystems [5–7]. However, structure and function may be maintained in ecosystems that have recently lost key species through the actions of so-called redundant and/or compensatory species; those that perform a similar ecological role [8] or can partially or solely compensate for the ecological role of declining or locally extinct species [9, 10], respectively. For example, previous studies have shown that carnivorous whelks (*Nucella* spp.) can play a role in stabilizing community structure by compensating for reductions in predation when the keystone predatory sea star *Pisaster ochraceus* is absent from rocky shore ecosystems [10]. Understanding the potential redundancy or compensatory role of such species in natural communities has become increasingly important as human activities have drastically altered ecosystems via habitat destruction [11, 12], the introduction of non-native species [13, 14], and the spread of disease [15, 16].

In nearshore coastal marine systems along the west coast of North America, sea star wasting disease (SSWD) has been decimating local sea star populations since an epidemic outbreak in 2013, impacting, in particular, the keystone predator *P*. *ochraceus* [16]. *P*. *ochraceus* plays an important role in maintaining a high level of diversity within rocky intertidal communities on the west coast through their consumption of a dominant space-holding mussel, *Mytilus californianus* [4]. By preying on *M*. *californianus*, *P*. *ochraceus* controls the mussels’ lower vertical distributional limit, where it would otherwise outcompete inferior space-holding competitors [4]. Thus, the consumptive effects of *P*. *ochraceus* drive distinctive patterns of both zonation and diversity along a vertical tidal elevation gradient. With the recent decimation of regional and local *P*. *ochraceus* populations, major community- and ecosystem-level consequences are expected; in particular, many primary space-holding species becoming scarce or absent as low zone communities switch to an alternative state dominated by mussels [17].

An open question is how widespread along the coast the community and ecosystem effects of SSWD will be. Menge et al. [17] recently hypothesized a ‘mosaic’ response to the SSWD outbreak, in which the low intertidal zone will become dominated by *M*. *californianus* at some sites but not at others. More specifically, mussels might not come to dominate in wave-protected areas of the coastline where alternative predators, like crabs, maintain influence over, or become more influential in affecting, intertidal community structure. Such redundancy and/or compensation could help maintain patterns of zonation and biodiversity along the vertical tidal elevation gradient.

Little work has been done on the potential role of crabs as predators on mussels in northeast Pacific rocky reef ecosystems, but see [18]. It has long been established that crabs can be an important driver of mortality and the intertidal zonation of mussels on wave-protected rocky reefs in Atlantic ecosystems [19–23] and we hypothesize that they are likely to play a similar ecological role on Pacific rocky reefs, particularly in wave-protected locations. For example, large cancrid crabs, like *Cancer productus*, have been shown to migrate from subtidal regions into the intertidal zone to feed, and are thought to limit the vertical distribution of littorine gastropods [24]. Further, on a per capita basis, large cancrid crabs can inflict a much greater degree of mortality on intertidal mollusc populations over a given time than *P*. *ochraceus* [25, 26]. Thus, crabs, in sufficient numbers, may be able to control the lower distributional limits of *M*. *californianus* in some regions and exert some control over community structure and diversity in intertidal systems.

In this study, we combined predator removals and predator exclusion cages in a field experiment aimed at examining the role of crab predation and other factors (temperature and wave force) on *M*. *californianus* mortality in the absence of *P*. *ochraceus*. Our goal was to assess the potential for crabs to play a similar predatory role as *P*. *ochraceus* in wave-protected areas of the west coast where *P*. *ochraceus* abundance is historically low and has been further decimated by SSWD. We hypothesize that in areas of high crab abundance, and in the absence of *P*. *ochraceus*, crab predation would be an important source of mortality on *M*. *californianus* in the low intertidal zone.

## Methods

### Study site

**Fig 1:**
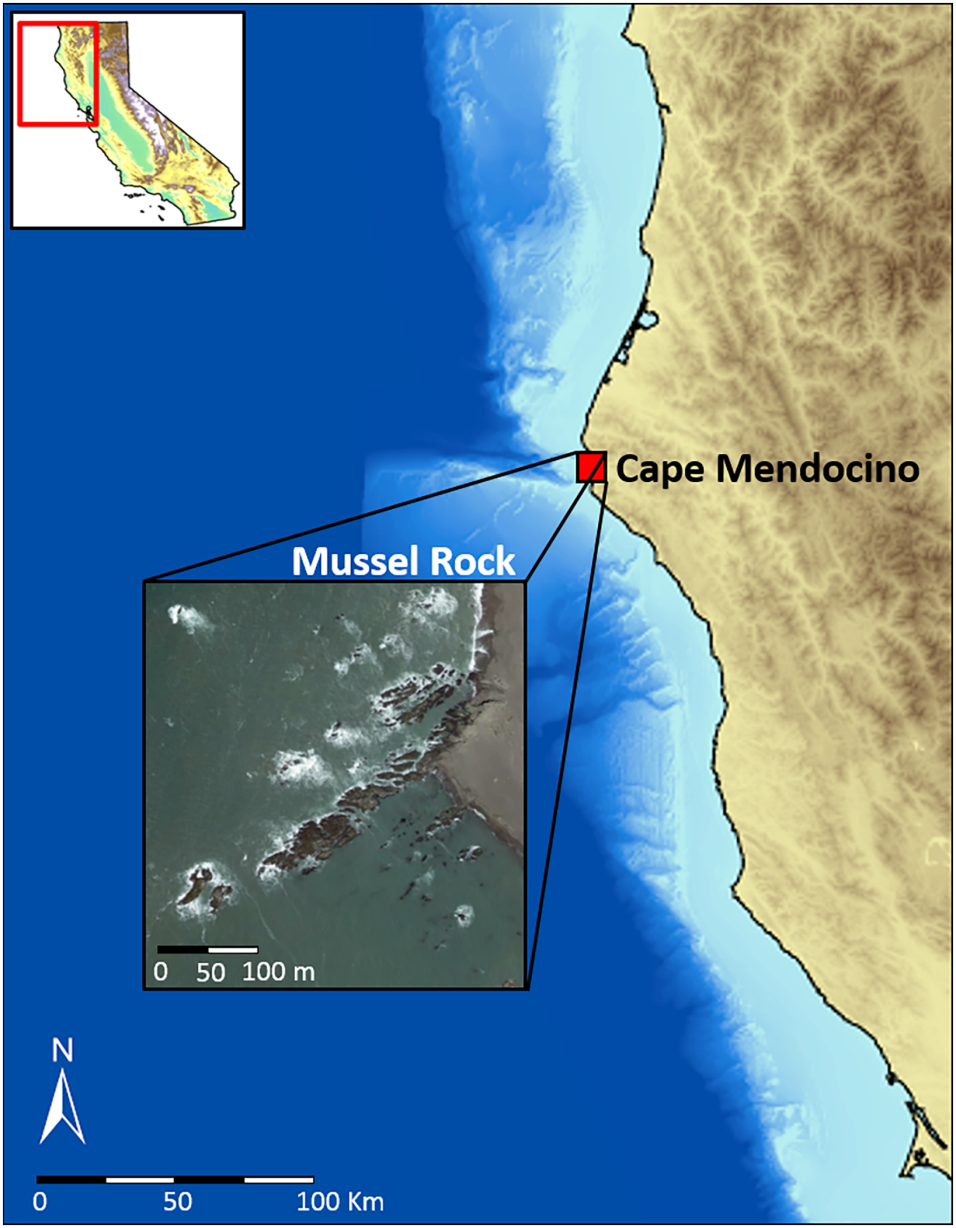
Location of field study site on Cape Mendocino, on the northern coast of California. Partially wave-protected rocky bench just southeast of Mussel Rock, where experimental cages, wax snail models, and environmental loggers were deployed (inset).

We did our experiments on a partially wave-protected bench just southeast (40°20’51.13” N, 124°21’51.87” W) of Mussel Rock, an exposed rocky reef along the northern coast of California on Cape Mendocino, during the summer of 2016. Our site consisted of a large rocky bench flanked on the north and south by small boulders and cobbles, that transition into stretches of sandy beach. The bench is partially protected from strong waves by a larger rocky bench to the north (Fig 1). This site therefore provides suitable habitat for both mussels (stable mid-zone bench) and crabs (wave-protected small boulder and cobble refuges), and is representative of other sites that are non-uniformly distributed among stretches of sandy beach and wave-exposed headlands along the northern California coastline. Mussel beds on this reef are located along the upper edge and on top of large rocky benches where they share space with macroalgae (*Pelvitiopsis limitata* and *Endocladia muricata)*. At lower elevations along, and adjacent to, these benches a variety of fleshy (*Alaria*, *Egregia*, *Fucus*, *Laminaria*, *Pyropia*, and *Saccharina spp*.) and coralline (*Corallina* and *Calliarthron spp*.) algae and bare boulders dominate. We observed a relatively low abundance of *P*. *ochraceus* compared to other localities in Northern California [27] and a noticeably high abundance of predatory crabs (*Romaleon antenarrium* and *C*. *productus*), making this locality ideal for investigating the potential for predatory crabs to control mussel distribution in the absence of *P*. *ochraceus*.

### Mussel bed characteristics

To quantify the distribution, abundance, and size structure of mussels at our study site, we quantified the percent cover and size distribution of *M*. *californianus* along a tidal elevation gradient. *M*. *californianus* is a dominant space-holder and habitat provider in the intertidal zone of rocky shores in the Eastern North Pacific that profoundly affects community diversity [28, 29]. We quantified percent cover of mussels within twenty-four 0.125 m^2^ quadrats at 5 tidal elevations (0.7, 1.2, 1.7, 2.2, & 2.7 m above MLLW) along a 40 m transect. Tidal elevations were measured with a laser level (CST/Berger LM30), using a reference point obtained by observing still tidal elevation on multiple days. We also determined the size frequency distribution of mussels by measuring the total shell length (mm) of all mussels within each of 13 haphazardly placed quadrats (0.125 cm^2^) from high (~2.7 m) tidal elevations at our site, where mussel cover was highest. Size frequency data was used to determine a realistic and ecologically relevant mussel size range to include in our experimental manipulations (see ‘*Caging experiments*’ below).

### Sea star size distribution, abundance, & removal

Prior to installing our experiments, we manually removed all *P*. *ochraceus* from our study site. During weekly visits to our site during the experiments we also removed any sea stars that had subsequently invaded our study area. Sea stars were measured for radius (distance between the center of the aboral surface of the central disc and the distal end of the longest arm), categorized as either adults or juveniles (<15 cm diameter) [17], and relocated to an adjacent reef, 60 m north of our site. We removed a total of 34 sea stars (3 adults and 31 juveniles) from our study site over the course of the two experiments. Two of the three adult *P*. *ochraceus* (average radius 167.13 mm ± 10.9) that were removed from our site were found on the surrounding boulders adjacent to the bench, but not on the bench or within the mussel beds themselves. Juveniles (average radius 30.20 mm ± 17.6) were found taking refuge within and along the periphery of mussel beds, but were likely too small to consume the majority of mussels that occupy these beds (average shell length 28.46 mm ± 0.82).

### Caging experiments

For experiment 1 we established 12 experimental blocks at our site; 6 at low tidal elevations (average elevation 0.7 m ± 0.07), 6 at high tidal elevations (average elevation 2.7 m above MLLW ± 0.19). Within each block, we established a pair of 15 x 15 cm mussel plots. At high elevations, these plots were established in naturally occurring mussel beds by removing existing mussels from a 3 cm margin around the edge of each plot. After we established all pairs of 15 x 15 cm mussel plots at high elevations, visible mussels within each plot were counted and the average number of mussels within each plot was used to determine the number of mussels transplanted to low tidal elevations. Counting only visible mussels in these plots prevented us from accurately enumerating any newly recruited mussels in our plots, as many of these recruits are hidden within the matrix of larger mussels in a given plot. However, doing so allowed us to keep the mussel plots intact and relatively undisturbed. Further, although we likely underestimated the actual number of mussels present within our plots, the number of visible mussels does provide us with a mussel density that falls within the natural range at our site. At low elevations, plots were established by transplanting 66 mussels (mean shell length = 28.46 mm SD ± 8.2) to plots (15 x 15 cm) on rocks cleared of any sessile invertebrates and any macro- and microalgae. Transplanted mussels were enclosed in plastic (Vexar^™^) mesh (0.4 cm^2^ openings) cages that were firmly attached to the substrate using drywall hangers with marine epoxy and stainless-steel screws and left for 10 days to allow for mussel byssal thread attachment before the start of the experiment. After 10 days, one cage from each pair of plots within each low elevation block was removed, and at high elevations, we fitted one plot from each pair with a cage; giving us one ‘caged’ and one ‘uncaged’ treatment plot within each block at low and high elevations.

For experiment 2, we used the same general procedure outlined in experiment 1, but made some alterations to control for the effects of transplantation. We again established 12 spatial blocks (6 at low elevations and 6 at high elevations), and we again cleared 15 x 15 cm patches of all sessile organisms and transplanted 66 mussels to each plot, at the low tidal elevation. However, instead of using naturally occurring 15 x 15 cm mussel patches at high elevations, we also transplanted mussels to cleared plots at high elevations; just as with the low elevation mussel transplants. Transplanting mussels to high tidal elevations where we did not expect crab predation to occur [24] allowed us to control for any effects of transplantation on mussel survival. Wave exposure can be severe at this site and in experiment 1 we observed a great deal of deformation and damage to cages at high tidal elevations. Although this was not the case at low elevations, we wanted to be confident that mussel mortality at low elevations was not wave-induced, so we also added a third ‘partial cage’ treatment, in which cages were open on the top to allow for crabs to feed but had a ‘fence’ to reduce water flow similar to that inside the full cage [30] and thus loss of mussels due to excessive flow. During pilot experiments, transplanted mussel mortality at high elevations was high in full cages, indicating that even full cages did not provide sufficient protection against the high flow environments there, so we therefore excluded partial cage treatments at high elevations. As in experiment 1, all transplanted mussels were given 10 days to securely attach to substrate before the start of the experiment. One pair of plots at high elevations was excluded from the data analysis due to severe dislodgment and the loss of the cage due to extreme wave forces.

At the end of each experiment, mussel survival was quantified by counting the number of intact and living mussels remaining within each plot after 16 days for experiment 1 and 46 days for experiment 2.

### Temperature & wave force

We quantified differences between low and high tidal elevations for two abiotic factors known to influence mussel mortality, temperature [31, 32] and wave force [31, 33]. This was done to aid in attributing differences in mussel mortality between tidal elevations to potential causal factors in our experimental manipulations. To determine whether temperature varied between low and high elevations, we used biomimetic mussel models (‘robo-mussels’) to measure the body temperature experienced by mussels in our experimental plots [32]. Robo-mussels were constructed by wrapping iButton^™^ (DS1921G-F50) temperature loggers in parafin wax and then encasing them in silicone within the valves of empty mussel shells, which were then glued shut with cyanoacrylate adhesive. One robo-mussel was placed between each of 5 pairs of experimental plots at low and high tidal elevations for a total of 10 robo-mussels for each caging experiment (See experiment 1 & 2 below). During each experiment, each iButton^™^ was programed to log internal mussel temperature every ten minutes for two weeks. Dynamometers, which measure maximum wave force [34], were also deployed between each of the 5 pairs of experimental plots at low and high tidal elevations for a total of 10 dynamometers for each experiment. Dynamometers were calibrated following methods in [34]. Over the course of both experiments, dynamometers were checked every 14–16 days to assess maximum extension between low and high elevations.

### Predation on wax snail replicas

To confirm whether crab predation was responsible for the loss of uncaged mussels at each tidal elevation, during experiment 2, we deployed wax snail replicas near our experimental blocks at both low and high elevations. Wax snail replicas were used because they have been shown to be an effective method to quantify crab predation, as the frequency of attacks on wax replicas are easily quantified and attributed to crabs [35] and also strongly correlated with crab scar repair frequency on the shells of living snails [35]. Four wax snail replicas (modeled after the whelk *Nucella ostrina*, a common inhabitant of our site) were bolted to each of twenty 8.5 x 8.5 cm plastic mesh screens, each of which were then bolted to the substrate in the vicinity of the caging experiment blocks at both low and high elevations for a total of 80 wax snail replicas. Crab attacks on snail replicas leave characteristic marks in the wax and were defined by the presence of either scarring marks attributable to the chelae or walking legs of crabs or by large portions of the replica missing due to crushing. When the entire wax replica and its attachment bolt were ripped away from the mesh (‘missing’), or when bolts were intact but missing their entire wax replica (‘incomplete’) we could not confidently attribute a particular causal agent to their demise, and so did not include them in our analyses of attack frequency. All arrays were deployed in the field for a total of 15 days before they were collected.

### Laboratory feeding assays

To quantify the difference between feeding rates of crabs and sea stars when consuming mussels and to determine how much more risk predatory crabs pose to mussel populations in terms of per biomass predation rate, we conducted a laboratory predation experiment in which both predatory crabs (*R*. *antenarrium*, the most commonly observed molluscivorous species at our site) and *P*. *ochraceus* were offered mussels (*M*. *californianus*) as prey. Both species of predators and mussels were collected from rocky reef habitat near the Telonicher Marine Laboratory in Trinidad, CA (41°03’1.88” N, 124°8’49.81” W). We offered 15 mussels of a range of shell lengths (25–60 mm) to each of 6 *R*. *antenarrium* and 6 *P*. *ochraceus*. *M*. *californianus* of these shell lengths are well within the range normally attacked by *P*. *ochraceus* of the sizes used in our experiment [28] and are capable of being crushed and consumed by *R*. *antennarium* (authors’ personal observation). Predator weights were kept as similar as possible in an effort to standardize biomass between treatments (mean *R*. *antennarium* wet mass = 172.2 g SD ± 88.9; mean *P*. *ochraceus* wet mass = 194.6 g SD ± 15.3). All 15 mussels were presented simultaneously to each predator. Predation trials took place in 2 closed-circuit sea tables (183 x 61 cm). Within each sea table predators were housed individually in separate plastic aquaria (30.5 x 19 x 20 cm) with flow-through seawater and covered with black tarp to prevent outside factors from affecting their motivation to feed. *R*. *antenarrium* were given 20 hours to forage; *P*. *ochraceus*, which took considerably longer to capture and consume prey, were given 168 hours to feed.

### Statistical analyses

All statistical analyses were done using R v3.2.2 (R Core Team 2015). We analyzed the relationship between mussel abundance and tidal elevation with an exponential regression, and used Welch’s two sample t-tests to assess differences in temperature and wave force between low and high tidal elevations.

Mussel mortality data from experiments 1 and 2 were converted into the mean number of mussels that were lost per day. To test the relative importance of caging and tidal elevation on mussel mortality we compared a set of generalized linear mixed models (*lme4* package) [36] that included the fixed factors of caging alone, elevation alone, and both together, including their interaction, along with a random factor for plot. We used two statistical approaches to compare models: (1) we used likelihood ratio tests (analysis of deviance) to do pairwise comparisons of full models and reduced models. The results of this comparison state whether additional variables in the model account for enough variance that one can reject the null hypothesis that the coefficients for these variables are zero. (2) An information theory approach to determine how well the data support each model. Specifically, we used Akaike’s Information Criterion with a second order correction (AICc) to assess the best-fit model (*AICcmodavg* package) [37]. All data from partial caged treatments implemented during experiment 2 were excluded from our analysis.

We used a Fisher’s exact test to determine whether the frequency of attacks on wax snail replicas was independent of tidal elevation. A Welch’s two-sample t-test was used to determine whether per biomass consumption rates of mussels differed between *R*. *antennarium* and *P*. *ochraceus* in laboratory feeding assays.

## Results

### Mussel bed characteristics

**Fig 2:**
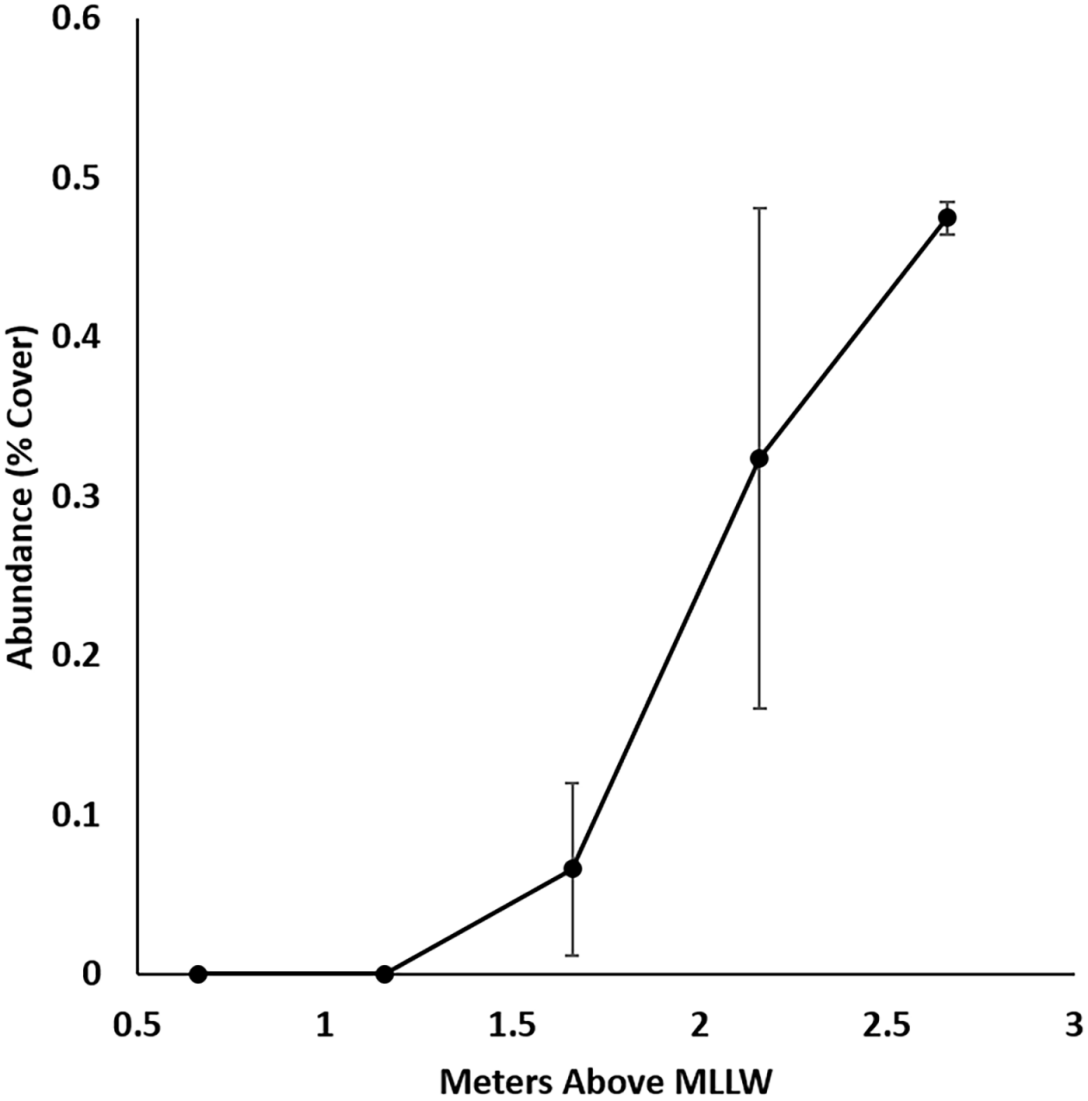
Mean (± 1 SE) abundance of *Mytilus californianus* across a tidal elevation gradient.

Mussel cover at our study site increased exponentially across the range of tidal elevations surveyed (0.7 to 2.7 m) at our site (y = 0.843e^0.212x^, *R*^2^ = 0.89, *P* = 0.019; Fig 2). Mussels were located along the tops and edges of the bench. Using the average percent cover from our quadrat survey, we estimated that mussels occupy roughly 24 percent of the 472 m^2^ sampling area that we surveyed. At the highest tidal elevations, where mussel cover was the greatest, mean mussel cover was 48 percent (SD ± 2.5). Mussel cover decreased to zero between 1.2 and 0.7 m above MLLW (Fig 2). The average total shell length of mussels from high (~2.7 m) tidal elevations was 28.46 cm ± 8.2.

### Caging experiments

**Fig 3:**
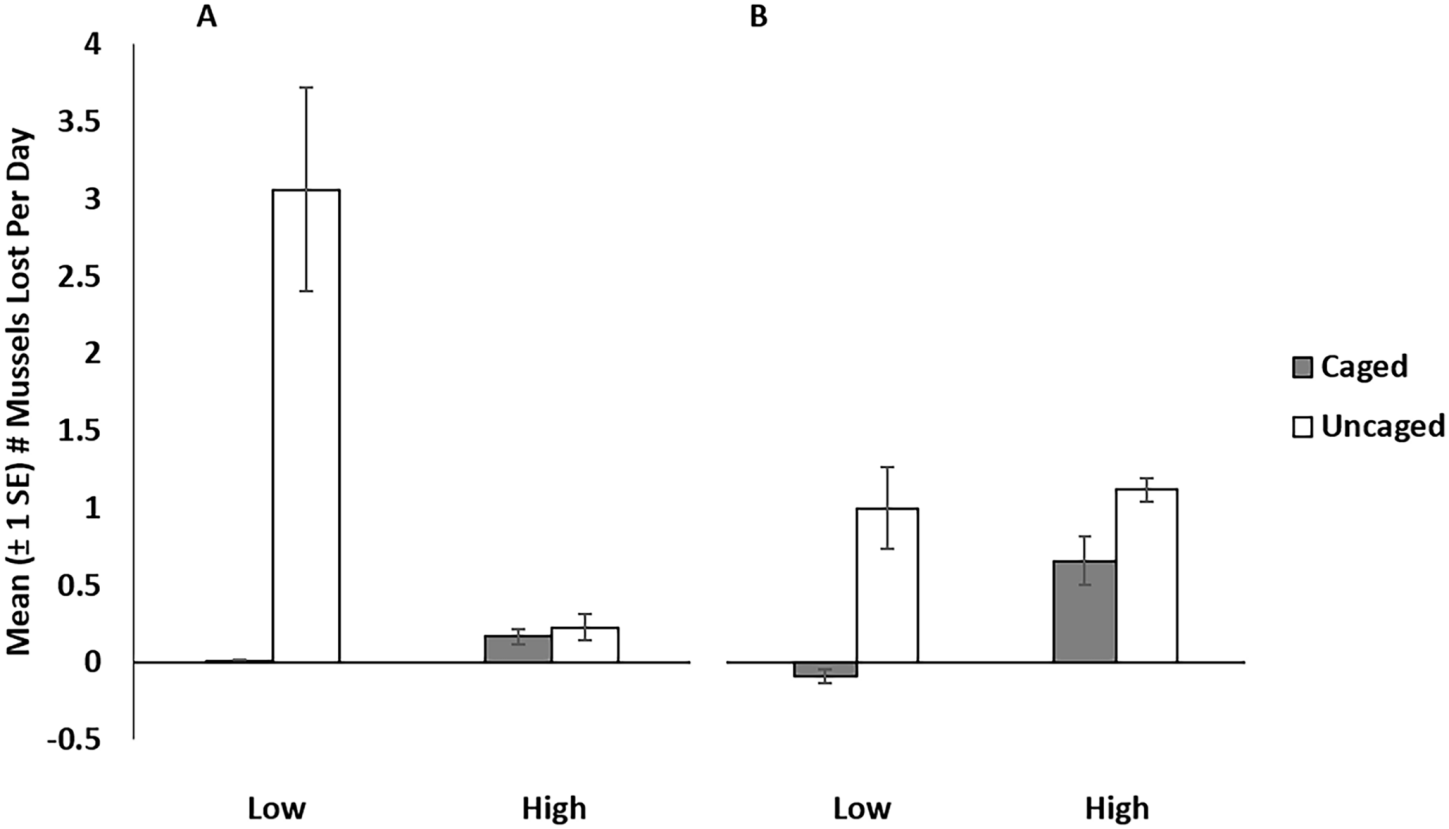
Mean (± 1 SE) number of *M*. *californianus* lost per day in caged and uncaged plots in (A) low tidal elevation reference plots (Low) and high tidal elevation transplant plots (High) in Experiment 1; and in (B) low and high tidal elevation transplant plots.

In experiment 1, analysis of deviance (Table 1A) showed a significant effect of caging (*P* = 0.001), tidal elevation (*P* = 0.004), and a significant effect of their interaction (*P* < 0.001) on mussel mortality. At high tidal elevation, 98 percent (SD ± 1.23) of mussels survived in caged treatments, while 97 percent (SD ± 2.25) survived in uncaged treatments (Fig 3A). At low tidal elevations, 100 percent (SD ± 0.62) of mussels survived in caged treatments, while 26 percent (SD ± 39.3) survived in uncaged treatments (Fig 3A). Comparison of AICc scores revealed that the full model, including the interaction between caging treatment and tidal elevation provided the best explanation for variation in mussel loss in Experiment 1. Inclusion of the caging * elevation interaction term resulted in an unambiguously superior, more informative model than the reduced models, even after penalizing for additional terms. The Akaike weight of the full model was 1 and none of the reduced models were within 10 AICc units of the full model (Table 2A; see Supporting information (S1A Table) for the results for each of the factors in the best model based on the model comparison).

**Table 1:** Analysis of deviance for generalized linear mixed models fitted to mussel mortality data for (A) experiment 1 and (B) experiment 2.

Model	Test	Df	Dev.	χ^2^	χ^2^ Df	*P*(χ^2^)
A. *Experiment 1*						
Full		6	54.18			
Cage+Elevation+Plot	-Cage*Elevation	5	70.80	16.61	1	<0.001
Cage+Plot	-Elevation	4	78.88	8.08	1	0.004
Elevation+Plot	-Cage	4	81.190	10.40	1	0.001
B. *Experiment 2*						
Full		6	64.74			
Cage+Elevation+Plot	-Cage*Elevation	5	68.19	3.45	1	0.063
Cage+Plot	-Elevation	4	72.09	3.91	1	0.048
Elevation+Plot	-Cage	4	89.41	1	21.23	<0.001

Model: predictor variables in the model. Test: predictor variable excluded from the full model. Df and Dev: degrees of freedom and deviance of the model. χ^2^ df represents the difference excluding the tested variable. The probability column indicates the significance of the deviance portion explained by each model term.

**Table 2:** Comparison of full and reduced models explaining mussel mortality in (A) Experiment 1 and (B) Experiment 2.

Candidate Model	K	AICc	Δ AICc	AICc Wt	Log Likelihood
A. *Experiment 1*					
Cage*Elevation+Plot	6	72.91	0.00	1.00	-27.98
Cage+Elevation+Plot	5	84.84	11.94	0.00	-35.76
Cage+Plot	4	89.29	16.39	0.00	-39.59
Elevation+Plot	4	91.41	18.51	0.00	-40.65
B. *Experiment 2*					
Cage*Elevation+Plot	6	81.34	0.00	0.47	-32.05
Cage+Elevation+Plot	5	82.25	0.90	0.30	-34.36
Cage+Plot	4	82.84	1.50	0.22	-36.31
Elevation+Plot	4	98.65	17.31	0.00	-44.22

K is the number of parameters in the model.

In experiment 2 we found no significant difference between partial cages and uncaged treatments at low elevation (*F*_1,10_ = 51, *P* = 1.00) and therefore omitted the partial cage treatment from our statistical analyses. Analysis of deviance (Table 1B) revealed significant effects of caging (*P* <0.001) and tidal elevation (*P* = 0.048) on mussel mortality and the effects of caging * elevation interaction on mussel mortality approaching significance (*P* = 0.063). At low tidal elevations, fifty-three percent (SD ± 27.2) of mussels survived in caged treatments and 21 percent (SD ± 13.1) survived in uncaged treatments (Fig 3B). At low tidal elevations, 106 percent (SD ± 7.55) survived (greater than 100 percent survival reflects presence of newly recruited mussels) in caged treatments and 29 percent (SD ± 45.4) survived in uncaged treatments (Fig 3B). Inclusion of caging effects resulted in the best, most informative models. AICc values were lower, and Akaike weights were higher for models including caging effects (Table 2B; see Supporting information (S1B Table) for the results for each of the factors in the best model based on the model comparison).

### Temperature & wave force

Average daily maximum robo-mussel temperatures at high tidal elevations (20.3, SD ± 1.80) were 50% greater and significantly different from those at low elevations (13.5, SD ± 1.11; *t*_(13.36)_ = -9.70, *P* <0.001). We also found a significant difference in maximum wave force between low and high tidal elevations (t_(3.75)_ = 293.25, *P*<0.001), where higher elevations experienced average maximum wave forces (9.1x10^3^ N/m^2^, SD ± 3.1) that were three times greater than those at lower elevations (3.1x10^3^ N/m^2^, SD ± 4.1).

### Predation on wax snail replicas

**Fig 4:**
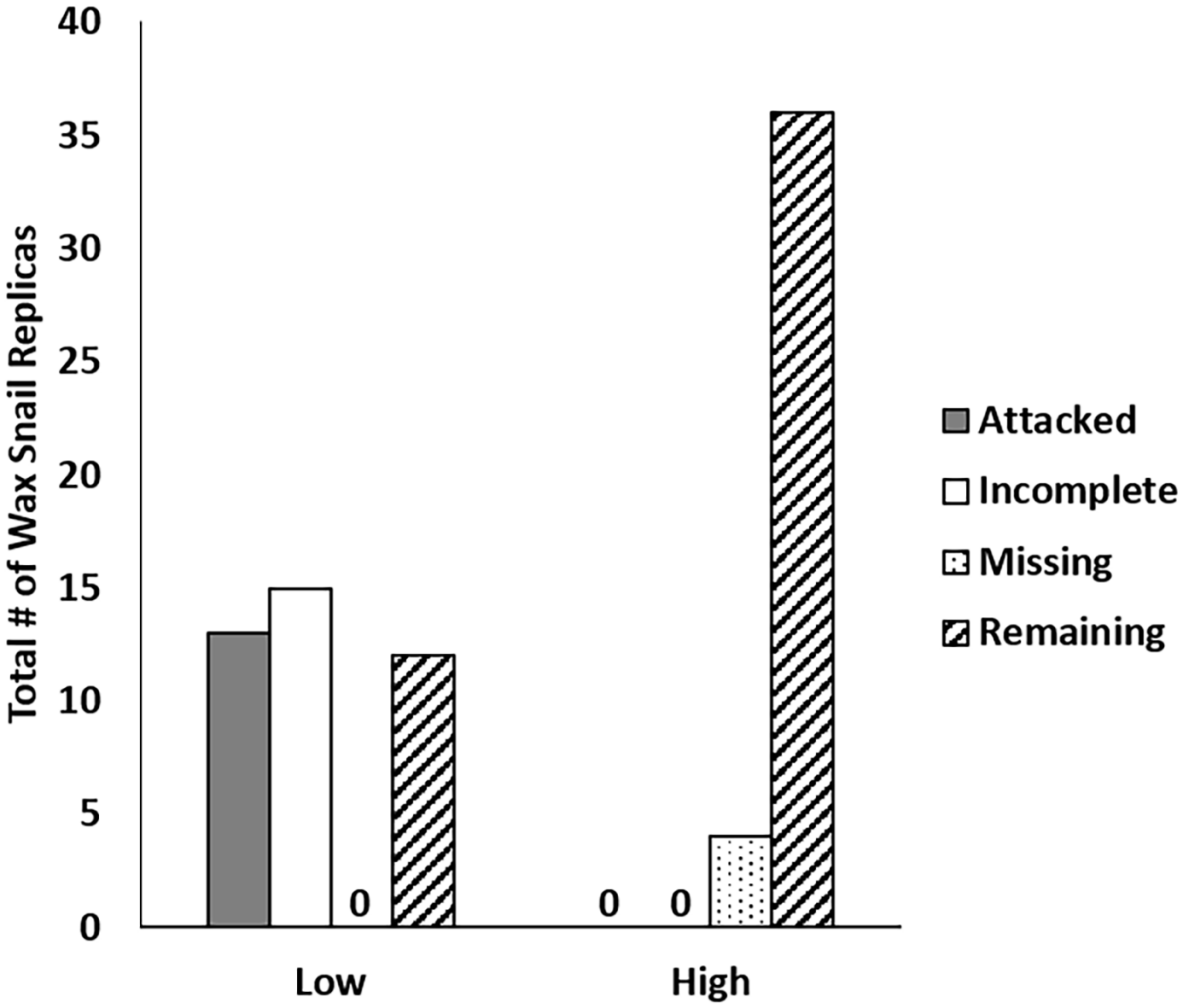
Frequency of attacked, incomplete, missing, and remaining wax snail replicas at low and high tidal elevations (‘0’ represents a frequency of zero).

Crab attack frequency on wax snail replicas depended on tidal elevation (*P*<0.001, 95% confidence intervals: 0.0, 0.136, odds ratio: 0; Fig 4). Replicas displaying confirmed crab attacks were only found at low elevations and no confirmed attacks were recorded at high elevations. We did observe a number of missing replicas (bolt and replica missing) at high elevations and incomplete replicas (bolt intact, but replica missing) at low elevations (Fig 4).

### Feeding assays

In laboratory feeding trials crabs consumed, per unit biomass, an average of 24.29 times more mussels per day than sea stars (t_(4.27)_ = 4.08, *P* = 0.012). Over the course of the 20-hour experiment crabs consumed a combined total of 25 mussels, while in 168 hours, sea stars consumed only a combined total of 10 mussels. On a per unit biomass basis, crabs consumed an average of 0.17 mussels per g of crab every day, while sea stars consumed only 0.007 mussels per g of sea star every day.

## Discussion

One potential consequence of the recent SSWD epidemic is that loss of the keystone predator *P*. *ochraceus* may shift the low intertidal zone of Pacific Coast rocky shores to an alternate community state dominated by mussels. However, recently Menge et al. [17] hypothesized that if alternative predators can compensate for the loss of sea stars by removing mussels at some locations, then the low intertidal zone may not become mussel-dominated at all locations. For example, mussels might gain low-shore dominance at wave-exposed areas, where alternative predators, like crabs, can be uncommon or have difficulty foraging [38]. However, mussels may not attain low-shore dominance in wave-protected areas where alternative predators, like crabs, are more numerous and influential [39]. Our results provide strong evidence that predatory crabs appear capable of causing significant mortality to mussels at low tidal elevations the absence of *P*. *ochraceus*.

**Fig 5:**
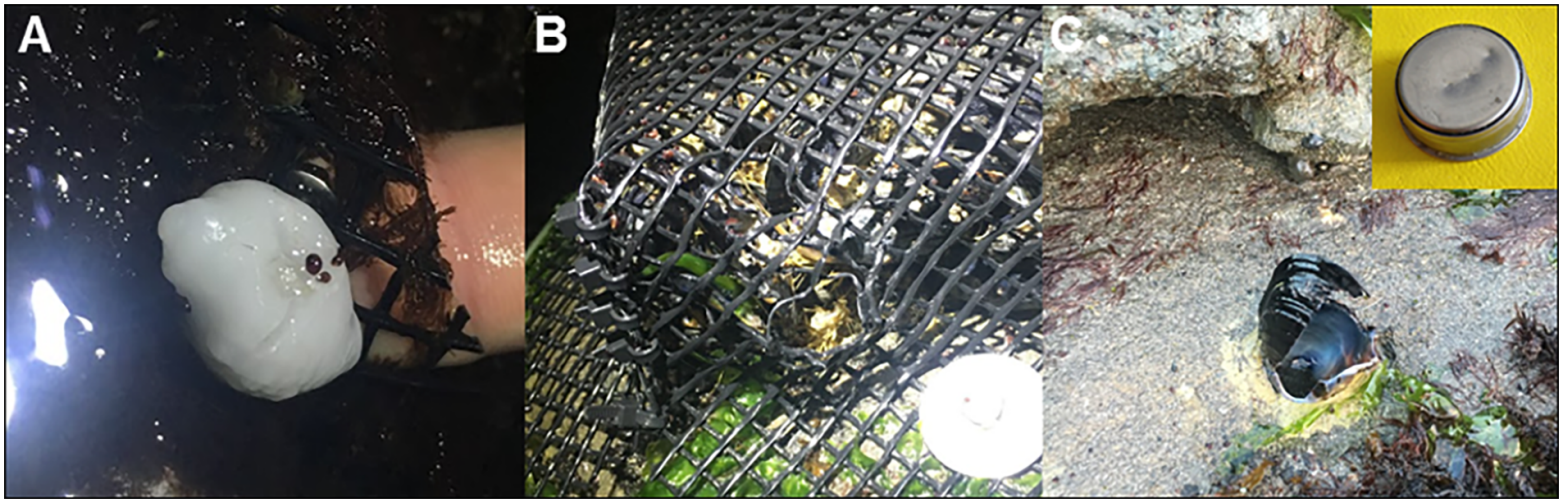
Photographic evidence of (A) crab attack on wax snail replica (B) crab-claw-induced damage to experimental cage, (C) crab attack on robo-mussel shell and temperature logger (inset).

Several lines of evidence indicate that the causal agent of mortality for mussels at low tidal elevations in our experiments was predation by crabs, rather than other predators. First, initial and subsequent removal of all *P*. *ochraceus* from our site prevented stars from feeding on experimental mussels, and we never noted any subtidal *P*. *ochraceus* at our site. The majority of substratum surrounding our experimental reef was small boulder and cobble bordered by sandy bottom, which offers little to no structural complexity for refuge or attachment sites for sea stars, and *P*. *ochraceus* is typically rare in these habitat types [40]. Further, even in the unlikely event that we missed any subtidal sea stars moving into our experimental plots, given their relatively slow consumption rates on mussels; it is highly unlikely that they could have accounted for the rapid mussel loss we observed in our experiments. Secondly, we did not find any carnivorous whelks that are capable of consuming *M*. *californianus* [10] at our site. *Nucella canaliculata*, an important consumer of *M*. *californianus*, is conspicuously absent from our study site, and although its congener *N*. *ostrina* is present, it is thought not to be capable of consuming the relatively thick-shelled *M*. *californianus*; and was only observed consuming barnacles at our site over the course of our two experiments. Further, we found no evidence of either attempted or successful drilling of mussels by whelks in caged or uncaged mussels, suggesting that whelks were not responsible for any of the mussel mortality we observed in our experiments. Thirdly, pile perch and striped surfperch, two species capable of consuming mussels [41–44], and are unlikely to consume *M*. *californianus* (D. Hankin, J. Jensen, and T. Mulligan, pers. com.). Fourthly, the presence of abundant broken shell fragments around the periphery of uncaged plots, residual byssal threads within uncaged plots and the high frequency of crab attacks on wax snail replicas at low elevations (Fig 5A) and the complete lack of attacks at high elevations, provide strong support that predatory crabs were the causal agent of mussel loss at low tidal elevations in our experiments. Lastly, we noted at least two instances where cages at low elevations in both experiment 1 & 2 were attacked by predatory crabs causing minor to moderate damage to the plastic mesh (Fig 5B) and two instances in which a low elevation robo-mussel had been attacked by a crab, which left a characteristic break in the mussel shell and claw marks in the temperature logger (Fig 5C). We observed no such damage to cages or robo-mussels in our high elevation plots.

Two potential sources of mussel mortality, thermal stress and dislodgement by wave forces, were not likely to have contributed to mussel loss at low tidal elevations in our experiments. Robo-mussels installed adjacent to our experimental plots at low tidal elevations indicated that average daily maximum mussel body temperatures in these habitats were no more than 3.7–4.2°C higher than the surrounding seawater temperature. Further, even though mussels transplanted to high elevations experienced body temperatures 1.5 times hotter than low elevation mussels, the range of temperatures our mussels experienced at both elevations resided well within the range of temperatures in which mussels can tolerate [45, 46], so it is not likely that thermal stress contributed to their demise.

Maximum wave forces (3.1 x 10^3^ N/m^2^) experienced by mussels at low tidal elevations at our site are characteristic of summer wave forces from exposed, west facing rocky reefs on the Pacific Coast [34, 47] where *M*. *californianus* predominates. Further, mussels in our partially caged plots, which could be accessed by crabs but were buffered from flow by a ‘fence’, did not show a reduction in loss relative to mussels in uncaged plots. In contrast, mussels transplanted to high elevation plots experienced maximum wave forces that were three times stronger than those experienced by mussels at low elevations and these mussels did experience high mortality both inside and outside of cages, which were often severely deformed, and in one case completely dislodged. If dislodgement by wave forces were responsible for mussel mortality at low elevations we would have observed similar cage deformation and high mussel mortality within caged plots, which we did not.

In combination with the results of our field experiments, the results of our feeding assays suggest the possibility that predatory crabs could have large effects on the abundance of intertidal *M*. *californianus* populations, and subsequently, rocky shore community structure and ecosystem function [4, 29]. Per capita, crabs can consume many more mussels per hour than sea stars, and if given the same amount of time to consume mussels as sea stars in our feeding assays, they would have consumed a combined total of approximately 210 mussels, translating to an estimated 1.25 mussels consumed per crab every hour. Therefore, in sufficient numbers, crabs could greatly deplete mussel populations. Further, the rapid, and relatively high mortality of uncaged mussel at lower tidal elevations in our experiments also suggest that crab predation could control the lower distributional limit of *M*. *californianus*. At our study site, where *P*. *ochraceus* is relatively rare [27], the distribution of mussels does not extend into the low intertidal zone or the subtidal zone, where adult *R*. *antennarium* and *C*. *productus* are abundant (authors’ personal observation) and risk of predation from these highly mobile predators should be higher [24]. Because predatory crabs aggregate to patches of high prey density, even in ephemerally favorable habitats like the high intertidal zone [24, 48], the spatial component of crab predation risk on mussels may be more difficult to predict than for slow moving, low shore species like *P*. *ochraceus*, which have more well-defined foraging zones [24]. Nevertheless, in the absence of *P*. *ochraceus*, mussels may become more abundant in the lower, most accessible intertidal zone for crabs, which may result in crabs aggregating to forage in these lower, prey-rich habitats; subsequently limiting the lower vertical distribution of mussels.

With the recent outbreak of SSWD decimating local and regional sea star populations, one possible outcome is dramatic changes in species composition and zonation patterns of intertidal rocky reef communities [17]. More specifically, the reduction or elimination of local *P*. *ochraceus* populations could cause an increase in overall mussel abundance and a downward shift in their vertical distribution, leading to a low zone community dominated by mussels [4]. An alternative outcome, however, is that current community structure persists even in the absence of *P*. *ochraceus*, owing to the pre-existing (i.e., redundant) or compensatory effects of other predators [9, 17].

At present, it is not clear whether crabs are functionally redundant with sea stars (feed on mussels with the same intensity), are compensating for sea star reductions, or historically have had a stronger predatory effect than sea stars on mussels at our site. For crabs to compensate for *P*. *ochraceus* they would need to increase their predation rate on mussels in the absence of sea stars; either through increases in abundance or per-capita consumption rate of mussels. Currently, we do not have data to support either of these outcomes. Subsequently, we cannot rule out the possibility that crabs may have always had a stronger predatory effect than sea stars on mussels at our site, making *P*. *ochraceus* functionally redundant. *P*. *ochraceus* has historically low abundances at our site compared to other northeast Pacific locations [27] and it is not clear that they play a keystone role at our study site. Nevertheless, our results suggest that predators like large, mobile crabs like *R*. *antennarium* and *C*. *productus* could potentially prevent the downward vertical spread and increased abundance of mussels in a manner similar to, or perhaps more effectively than *P*. *ochraceus*; due to the crabs’ ability to consume mussels at a much faster rate and their relatively high abundance at lower tidal elevations in some locations [49]. In the wake of SSWD, alternative predators, such as molluscivorous crabs and lobsters, may therefore have a more profound effect on their environment than previously appreciated [17, 18, 24]; which may prove important for understanding the future of rocky shore community structure and functionality.

## Supporting information

S1 Data Set Percent cover of mussels at 5 tidal elevations along a 40 m transect. (CSV)

S2 Data Set Mussel mortality in caging experiment 1. (CSV)

S3 Data Set Mussel mortality in caging experiment 2. (CSV)

S4 Data Set Average daily maximum robo-mussel temperatures in experimental plots at low and high tidal elevations. (CSV)

S5 Data Set Maximum wave forces in experimental plots at low and high tidal elevations. (CSV)

S6 Data Set Consumption rates of crabs and sea stars feeding on mussels in laboratory feeding assays. (CSV)

S1 Table Results of analysis of variance (ANOVA) for each of the factors in the best model based on the model comparison in Table 2. (DOCX)
