# Supplementary material for: Can crabs kill like a keystone predator? A field-test of the effects of crab predation on mussel mortality on a northeast Pacific rocky shore
Source: PLoS One. 2017 Aug 24;12(8):e0183064. doi: 10.1371/journal.pone.0183064 (PMC5570281; doi:10.1371/journal.pone.0183064)
Supplement: S1 Table — (DOCX) [file pone.0183064.s007.docx]

**S4 Table. Results of analysis of variance (ANOVA) for each of the factors in the best model based on the model comparison in Table 2.**

A. Experiment 1

Df SS MS *F P*

Elevation 1 0.270 0.270 22.42 <0.001

Cage 1 0.514 0.514 42.63 <0.001

Plot 10 0.142 0.014 1.18 0.401

Elevation:Cage 1 0.448 0.448 37.11 <0.001

Residuals 10 0.121 0.012

B. Experiment 2

Df SS MS *F P*

Elevation 1 0.676 0.676 11.15 0.009

Cage 1 1.429 1.429 23.58 <0.001

Plot 10 0.318 0.032 0.53 0.835

Elevation:Cage 1 0.345 0.345 5.69 0.041

Residuals 9 0.546 0.061
